# Prevalence of mental disorders and their associations with age at diagnosis and time since diagnosis of nasopharyngeal cancer

**DOI:** 10.3389/fpubh.2024.1469001

**Published:** 2024-12-03

**Authors:** Wen-Xuan Wang, Yi-Shan Wu, Li-Ping Qi, Anise M. S. Wu, Ying-Ying Zhu, Wei-Jie Gong, Shan-Shan Guo, Yi-Jun Hua, Dong-Hua Luo, Qiu-Yan Chen, Yan-Qun Xiang, Jin-Xin Zhang, Hai-Qiang Mai, Ji-Bin Li

**Affiliations:** ^1^School of Public Health, Sun Yat-sen University, Guangzhou, China; ^2^Department of Clinical Research, Sun Yat-sen University Cancer Center, Guangzhou, China; ^3^Department of Nasopharyngeal Carcinoma, Sun Yat-Sen University Cancer Center, Guangzhou, China; ^4^State Key Laboratory of Oncology in South China, Guangdong Key Laboratory of Nasopharyngeal Carcinoma Diagnosis and Therapy, Sun Yat-sen University Cancer Center, Guangzhou, China; ^5^Department of Radiation Oncology, Sun Yat-sen University Cancer Center, Guangzhou, China; ^6^Department of Psychology, Faculty of Social Sciences, University of Macau, Taipa, Macao SAR, China; ^7^Centre for Cognitive and Brain Sciences, Institute of Collaborative Innovation, University of Macau, Taipa, Macao SAR, China; ^8^Clinical Research Design Division, Clinical Research Center, Sun Yat-sen Memorial Hospital, Sun Yat-sen University, Guangzhou, China; ^9^Department of Family Medicine, Shenzhen University Medical School, Shenzhen University, Shenzhen, China

**Keywords:** nasopharyngeal carcinoma, depression, anxiety, sleep disorders, age at diagnosis, time since diagnosis

## Abstract

**Background:**

Despite advancements in cancer treatment, understanding the long-term mental health implications for nasopharyngeal carcinoma (NPC) survivors remains an underexplored area. This study aims to examine the prevalence of mental disorders and their correlations with age at diagnosis and time since diagnosis among NPC survivors.

**Methods:**

A total of 1872 NPC patients were surveyed from September 2020 to June 2021 in this cross-sectional survey. Logistic regression models were used to analyze the associations of age at diagnosis and time since NPC diagnosis with the risk of mental disorders. Additionally, the potential nonlinear trend between these factors was examined using restricted cubic splines. Analyses were conducted both overall and stratified by gender. Gender interaction was also examined.

**Results:**

The prevalences of depression, anxiety, and sleep disorders were 32.4, 33.2, and 61.5%, respectively. Age at NPC diagnosis was significantly associated with an elevated risk of depression (adjusted OR (aOR): 1.75 for 30–39 years old; 2.33 for 50–59 years old; 2.59 for ≥60 years old) and sleep disorders (aOR: 2.41 for 40–49 years old; 1.95 for 50–59 years old; 2.26, for ≥60 years old), compared to patients diagnosed with NPC at age < 30 years. Conversely, the risk of depression, anxiety, and sleep disorders exhibited negative associations with the time since diagnosis, compared to patients <3 months. Notably, significant nonlinear associations were observed between time since diagnosis and the risk of depression, anxiety, and sleep disorders, which showed an initial increase, with the highest risk occurring at approximately 3.0 (OR_max_: 2.7), 1.5 (OR_max_: 2.1), and 4.0 (OR_max_: 1.9) months since NPC diagnosis, followed by a gradual recovery to a lower risk level at around 12 months. No gender interactions were observed.

**Conclusion:**

The prevalence of mental disorders is notable among NPC survivors, showing a positive correlation with age at diagnosis while displaying a negative correlation with time since diagnosis, thus indicating the need for psychological support, especially within the initial several months following NPC diagnosis.

## Introduction

Nasopharyngeal carcinoma (NPC) is an epithelial cancer originating from the nasopharynx epithelium (1). Epidemiological assessments reported approximately 133,000 newly diagnosed cases of NPC in 2020, with more than 85.2% occurring in Asia, notably in Southern China in regions such as Guangdong and Guangxi provinces (2–6). Over the past two decades, advancements in early detection and treatment modalities have significantly enhanced the prognosis for NPC patients, with the five-year overall survival rate increasing to 94.6% for early-stage NPC patients while ranging from 78.3 to 88.9% for those in stages III-IV (7, 8), thus leading to a considerable population of patients transitioning into long-term survivorship.

NPC patients have been found to encounter various physical and mental health challenges at diagnosis, during treatment, and in the post-treatment period. For instance, the rates of depression, anxiety, and sleep disorders have been reported to significantly increase before, during, and after radiotherapy (9, 10). In addition, after radiotherapy, there could be a significant increase in the prevalence of anxiety, depression, and poor sleep quality compared to pre-radiotherapy levels (19.6% vs. 3.9%; 39.2% vs. 3.9%; 64.7% vs. 37.3%, respectively) (11). These mental problems contribute to emotional instability and maladaptive coping behaviors such as hopelessness and anxious preoccupation, ultimately diminishing health-related quality of life, and therapeutic adherence (12). Moreover, psychological distress can adversely reduce the survival prognosis of NPC patients, as it may compromise the effectiveness of treatment and increase the likelihood of disease progression (13, 14). Severe mental disorders may also increase the risk of suicidal ideation and behaviors (15, 16), thereby increasing healthcare costs due to elevated utilization of mental and primary healthcare services among NPC patients (17, 18).

As the number of long-term survivors among NPC patients grows, it becomes imperative to pinpoint when mental disorders pose the most significant risk to allow for timely and targeted psychological interventions. Psychological support and intervention could be highly beneficial for the mental health of cancer patients, helping to reduce their fear of cancer recurrence, improve mood, enhance pain perception and improve the emotional function aspect of health-related quality of life (19–21). However, the trajectory of mental disorders with both age at diagnosis and time since diagnosis remains unclear, hindering the development of tailored interventions.

Previous studies on NPC have predominantly focused on psychological issues at the time of diagnosis and during treatment phases (9, 10). These studies provide essential insights into the immediate psychological impact of NPC diagnosis and treatment; however, there is a notable gap in understanding the relationship between psychological distress and the time since NPC diagnosis. Limited research has explored how psychological well-being evolves, and distinct stages post-diagnosis may present unique psychological challenges and support needs. Addressing this gap is crucial for developing effective, long-term mental health interventions tailored to the evolving needs of NPC patients.

To bridge this knowledge gap, this study aims to investigate the prevalence of mental disorders (specifically depression, anxiety, and sleep disorders) and their associations with age at diagnosis and time since diagnosis among NPC survivors in South China, which is one of the most representative endemic regions for NPC worldwide. Findings will provide essential insights into identifying high-risk individuals and determining the optimal timing for interventions.

## Methods

### Study design and participants

This cross-sectional survey was conducted at our Cancer Center in southern China, which is one of the most representative NPC endemic areas of the world. Eligible patients were aged 18 years or older, pathologically confirmed with NPC, without cognitive impairment, and capable of self-administering questionnaires. Upon obtaining informed consent, eligible patients completed a structured questionnaire with the assistance of field workers. Patients were assured that their data would only be accessed by the researchers. A total of 2,268 patients were surveyed between September 2020 and June 2021, with 1872 of them completing the questionnaires and included in this study. We did not handle missing data and conducted analyses using available data directly. Consequently, there were differences in sample sizes for each mental disorder.

### Measurements

#### Depression and anxiety

Depression and anxiety were assessed using the Chinese version of the Hospital Anxiety and Depression Scale (HADS) (22), which comprises two 7-item subscales dedicated to measuring anxiety and depression, respectively. Each item is scored on a four-point Likert scale ranging from 0 to 3, resulting in a total score ranging from 0 to 21 for each subscale, whereby a higher score indicates a greater severity of anxiety or depression. In this study, Cronbach’s *α* coefficients were 0.81 for the anxiety subscale and 0.75 for the depression subscale, indicating satisfactory reliability. Depression and anxiety cases were defined as individuals with a subscale score of 8 or higher (22).

#### Sleep disorders

The Pittsburgh Sleep Quality Index (PSQI) was utilized to assess sleep disorders experienced over the past month (23), and its Chinese version has shown favorable overall psychometric properties (24). Comprising 18 items, the PSQI evaluates patients’ experiences across seven components: sleep latency, sleep duration, habitual sleep efficiency, subjective sleep quality, use of sleep medication, sleep disturbances, and daytime dysfunction. Each component is rated on a scale from 0 (no difficulty) to 3 (severe difficulty). A global score ranging from 0 to 21 is derived by summing the scores of the seven components, with higher scores indicating poorer sleep quality. Sleep disorders are defined as individuals with PSQI global scores exceeding five (23, 24). In this study, the Cronbach’s *α* coefficients for the seven components were 0.76, indicating satisfactory internal consistency.

#### Demographic and clinical covariates

The sociodemographic characteristics collected through the questionnaire surveys included gender, age at pathological diagnosis, body weight, height, living location, marital status, education level, monthly family income, family history of cancers among first-degree relatives, smoking status, alcohol use, financial debt resulting from anti-cancer treatment, and weekly physical activity level in the past year. Body mass index (BMI) was computed as weight (in kilogram [kg]) divided by the square of height (in meter [m]), then categorized into three groups according to World Health Organization guidelines: underweight (BMI <18.5 kg/m^2^), normal weight (BMI ranging from 18.5 to 24.9 kg/m^2^), and overweight or obesity (BMI ≥25.0 kg/m^2^). Age at diagnosis, clinical stage, treatment modalities, recurrence, and metastatic status variables were extracted from the patient’s medical records. Time since NPC diagnosis was calculated as the interval in months from the date of NPC pathological diagnosis to the date of survey completion.

### Statistical methods

Continuous variables are presented as means with standard deviations (SD) or as medians with interquartile ranges (IQR) when appropriate. Categorical variables are shown as frequencies with proportions. The prevalences of depression, anxiety, sleep disorders, and their comorbidity were estimated and then further stratified by gender, along with their corresponding 95% confidence intervals (CIs).

Binary logistic regression models were applied to investigate the associations between age at diagnosis and time since NPC diagnosis with depression, anxiety, and sleep disorders. Adjustments were made for various factors, including gender, BMI, living location, marital status, educational level, monthly family income, family history of cancer in first-degree relatives, smoking status, alcohol use, financial debt due to anti-cancer treatment, physical activity level, clinical stage, treatment modalities, distant metastasis status, and recurrence status. Both age at diagnosis and time since diagnosis were considered as both continuous and categorical variables. Odds ratios (ORs) with corresponding 95% CIs were calculated. Gender interaction was also examined. Nonlinear trends of mental disorders with age at diagnosis and time since diagnosis were evaluated using restricted cubic spline regression models (25), with the median value as the reference. Analyses were conducted both overall and stratified by gender. No missing data handling method.

All statistical analyses were performed using the R software (version 4.3.3), and a two-sided *p* < 0.05 was considered statistically significant.

### Ethics

This study was approved by the institutional review board of our Cancer Center (IRB NO: B2020-203), and written informed consent was obtained from all participants. The authenticity of this article has been validated by uploading the key raw data onto the Research Data Deposit platform^1^ with the approval RDD number RDDA2024544536.

## Results

### Patients’ characteristics

The mean age of the patients was 44.1 years (SD: 10.7; range: 18.0–80.0). We observed that 70.6% were male, and 69.6% had normal weight. A family history of cancer in first-degree relatives was reported by 19.4% of patients, and 50.9% faced financial debt due to anti-cancer treatment. Furthermore, 35.9% had a history of smoking, 34.2% had a history of alcohol use, and 22.3% reported no physical activity in the past year.

Among the 1872 patients, 92.1% were diagnosed in clinical stages III-IV, and 64.3% were diagnosed with NPC between the ages of 30 and 49. Only 11.2% of patients survived more than 5 years. The median time since NPC diagnosis was 14.0 months (IQR: 3.0, 32.0). In addition, approximately 94.0% received intensity-modulated radiotherapy, 93.9% underwent chemotherapy, and 4.4% underwent surgery (Table 1). The associations between sociodemographic and clinical variables with the three mental disorders are shown in Supplementary Table 1.

**Table 1:** Baseline characteristics.

	Total (*N* = 1872) *n* (%)	Sample for depression analysis (*N* = 1794) *n* (%)	Sample for anxiety analysis (*N* = 1820) *n* (%)	Sample for sleep disorders analysis (*N* = 1,419) *n* (%)
Social-demographic variables				
Gender				
Male	1,322 (70.6)	1,265 (70.5)	1,284 (70.5)	993 (70.0)
Female	550 (29.4)	529 (29.5)	536 (29.5)	426 (30.0)
Age, years, Mean ± SD	44.1 **±** 10.7	44.0 **±** 10.7	44.0 **±** 10.7	44.1 **±** 10.7
BMI, kg/m^2^, Mean ± SD	21.9 **±** 3.30	21.9 **±** 3.29	21.9 **±** 3.31	22.0 **±** 3.33
<18.5	272 (14.5)	261 (14.5)	268 (14.7)	200 (14.1)
18.5–24.9	1,302 (69.6)	1,250 (69.7)	1,263 (69.4)	988 (69.6)
≥25.0	298 (15.9)	283 (15.8)	289 (15.9)	231 (16.3)
Living location				
Urban	1,160 (62.0)	1,116 (62.2)	1,130 (62.1)	882 (62.2)
Rural	712 (38.0)	678 (37.8)	690 (37.9)	537 (37.8)
Marital status				
Single (i.e., Unmarried/divorced/separated/widowed)	273 (14.6)	262 (14.6)	267 (14.7)	201 (14.2)
Married/cohabiting	1,599 (85.4)	1,532 (85.4)	1,553 (85.3)	1,218 (85.8)
Education level				
High school or below	1,215 (64.9)	1,154 (64.3)	1,173 (64.5)	912 (64.3)
Junior college/Bachelor’s degree or above	657 (35.1)	640 (35.7)	647 (35.5)	507 (35.7)
Monthly family income, RMB				
<5,000	1,066 (56.9)	1,015 (56.6)	1,029 (56.5)	803 (56.6)
≥5,000	806 (43.1)	779 (43.4)	791 (43.5)	616 (43.4)
Family history of cancer in first-relatives				
No	1,508 (80.6)	1,444 (80.5)	1,465 (80.5)	1,132 (79.8)
Yes	364 (19.4)	350 (19.5)	355 (19.5)	287 (20.2)
Financial debt due to anti-cancer treatment				
No	920 (49.1)	879 (49.0)	893 (49.1)	696 (49.0)
Yes	952 (50.9)	915 (51.0)	927 (50.9)	723 (51.0)
Smoking status				
Never	1,199 (64.0)	1,147 (63.9)	1,164 (64.0)	910 (64.1)
Former	592 (31.6)	572 (31.9)	581 (31.9)	445 (31.4)
Current	81 (4.3)	75 (4.2)	75 (4.1)	64 (4.5)
Alcohol use				
No	1,231 (65.8)	1,179 (65.7)	1,192 (65.5)	934 (65.8)
Yes	641 (34.2)	615 (34.3)	628 (34.5)	485 (34.2)
Weekly frequency of physical activity				
Never	455 (24.3)	431 (24.0)	444 (24.4)	322 (22.7)
1 time	401 (21.4)	383 (21.3)	388 (21.3)	305 (21.5)
2–3 times	442 (23.6)	429 (23.9)	429 (23.6)	348 (24.5)
4–5 times	157 (8.4)	152 (8.5)	155 (8.5)	127 (9.0)
Almost everyday	417 (22.3)	399 (22.2)	404 (22.2)	317 (22.3)
Clinical variables				
Clinical stage				
I	43 (2.3)	42 (2.3)	40 (2.2)	34 (2.4)
II	105 (5.6)	103 (5.7)	104 (5.7)	79 (5.6)
III	937 (50.1)	900 (50.2)	913 (50.2)	715 (50.4)
IV	787 (42.0)	749 (41.8)	763 (41.9)	591 (41.6)
Age at diagnosis, years, Mean ± SD	42.0 **±** 10.7	42.0 **±** 10.7	42.0 **±** 10.7	42.0 **±** 10.6
<30	212 (11.3)	204 (11.4)	206 (11.3)	159 (11.2)
30–39	602 (32.2)	585 (32.6)	589 (32.4)	455 (32.1)
40–49	600 (32.1)	565 (31.5)	583 (32.0)	456 (32.1)
50–59	354 (18.9)	338 (18.8)	341 (18.7)	274 (19.3)
≥60	104 (5.6)	102 (5.7)	101 (5.5)	75 (5.3)
Survival time, months, Median (IQR)	14.0 (3.0, 32.0)	13.5 (3.0, 32.0)	13.0 (3.0, 32.0)	14.0 (3.0, 32.0)
<3	425 (22.7)	413 (23.0)	418 (23.0)	311 (21.9)
3–5	244 (13.0)	234 (13.0)	237 (13.0)	179 (12.6)
6–11	221 (11.8)	209 (11.7)	215 (11.8)	165 (11.6)
12–23	333 (17.8)	320 (17.8)	324 (17.8)	266 (18.7)
24–59	440 (23.5)	425 (23.7)	427 (23.5)	345 (24.3)
≥60	209 (11.2)	193 (10.8)	199 (10.9)	153 (10.8)
Intensity-modulated radiotherapy				
No	112 (6.0)	110 (6.1)	109 (6.0)	87 (6.1)
Yes	1760 (94.0)	1,684 (93.9)	1711 (94.0)	1,332 (93.9)
Chemotherapy				
No	115 (6.1)	111 (6.2)	111 (6.1)	87 (6.1)
Yes	1757 (93.9)	1,683 (93.8)	1709 (93.9)	1,332 (93.9)
Surgery				
No	1790 (95.6)	1714 (95.5)	1741 (95.7)	1,356 (95.6)
Yes	82 (4.4)	80 (4.5)	79 (4.3)	63 (4.4)
Distant metastasis				
No	1,606 (85.8)	1,539 (85.8)	1,564 (85.9)	1,213 (85.5)
Yes	266 (14.2)	255 (14.2)	256 (14.1)	206 (14.5)
Recurrence				
No	1720 (91.9)	1,645 (91.7)	1,670 (91.8)	1,316 (92.7)
Yes	152 (8.1)	149 (8.3)	150 (8.2)	103 (7.3)

BMI, Body mass index; NPC, Nasopharyngeal carcinoma; SD, standard deviation; IQR, interquartile range.

### Prevalence of mental disorders and comorbidity

The overall prevalences of depression, anxiety, and sleep disorders were 32.4% (95% CI: 30.3–34.7%), 33.2% (95% CI: 31.1–35.5%), and 61.5% (95% CI: 58.9–64.1%), respectively. The prevalences of anxiety (37.9% vs. 31.3%, *p* = 0.007) and sleep disorders (70.0% vs. 57.9%, *p* < 0.001) were significantly higher in females compared to males. The comorbidity prevalences involving depression and anxiety, depression and sleep disorders, as well as anxiety and sleep disorders, were 5.8% (95% CI: 4.7–7.0%), 8.8% (95% CI: 7.4–10.5%), and 9.4% (95% CI: 7.9–11.0%), respectively. The comorbidity prevalence involving all three mental disorders was 4.3% (95% CI: 3.3–5.5%) (Table 2).

**Table 2:** The prevalence of mental disorders and comorbidity.

Psychological disorders	All		Male		Female		*P* for gender
	Cases/*n*	Prevalence (95% CI)	Cases/*n*	Prevalence (95% CI)	Cases/*n*	Prevalence (95% CI)	
Depression	582/1794	32.4 (30.3–34.7)	397/1265	31.4 (28.8–34.0)	185/529	35.0 (30.9–39.2)	0.139
Anxiety	605/1820	33.2 (31.1–35.5)	402/1284	31.3 (28.8–33.9)	203/536	37.9 (33.8–42.1)	0.007
Sleep disorders	873/1419	61.5 (58.9–64.1)	575/993	57.9 (54.8–61.0)	298/426	70.0 (65.4–74.3)	<0.001
Depression and Anxiety	102/1765	5.8 (4.7–7.0)	63/1244	5.1 (3.9–6.4)	39/521	7.5 (5.4–10.1)	0.047
Depression and sleep disorders	122/1381	8.8 (7.4–10.5)	77/965	8.0 (6.3–9.9)	45/416	10.8 (8.0–14.2)	0.088
Anxiety and sleep disorders	130/1389	9.4 (7.9–11.0)	78/971	8.0 (6.4–9.9)	52/418	12.4 (9.4–16.0)	0.010
Depression, anxiety, and sleep disorders	58/1360	4.3 (3.3–5.5)	34/949	3.6 (2.5–5.0)	24/411	5.8 (3.8–8.6)	0.059

95% CI: 95% confidence interval.

The prevalence trends of depression, anxiety, and sleep disorders with age at NPC diagnosis and time since NPC diagnosis are shown in Supplementary Figures 1, 2, and the scores of depression, anxiety, and sleep disorders, along with seven components stratified by gender, are shown in Supplementary Table 2. Overall, it can be observed that female patients reported significantly higher scores compared to male patients.

### Associations between age at diagnosis and mental disorders

The risk of depression and sleep disorders was significantly associated with age at diagnosis. Compared to patients diagnosed with NPC at age < 30 years, the adjusted ORs (aORs) for depression were 1.75 (95%CI: 1.17–2.61; *p* = 0.007) in the 30–39 years group, 2.33 (95% CI: 1.48–3.67; *p* < 0.001) in the 50–59 years group, and 2.59 (95% CI: 1.46–4.58; *p* = 0.001) in the ≥60 years group. The aORs for sleep disorders were 2.41 (95% CI: 1.55–3.75; *p <* 0.001) in the 40–49 years group, 1.95 (95% CI: 1.21–3.14; *p =* 0.006) in the 50–59 years group, and 2.26 (95% CI: 1.18–4.31; *p =* 0.013) in ≥60 years group. Moreover, for every 10-year increase in age at diagnosis, the aORs were 1.22 (95% CI: 1.10–1.36; *p* < 0.001) for depression, 1.16 (95% CI: 1.04–1.29; *p* = 0.008) for anxiety, and 1.27 (95% CI: 1.12–1.43; *p* < 0.001) for sleep disorders. No significant interaction was observed with gender (Table 3). The results stratified by gender are presented in Supplementary Table 3.

**Table 3:** Association between age at diagnosis and depression, anxiety and sleep disorders.

	Case/*n*	Prevalence (95% CI)	Crude OR (95% CI)^a^	Adjusted OR (95% CI)^b^	*P* for gender interaction
Depression (*n* = 1794)					
Age at diagnosis, years					0.068
<30	49/204	24.0 (22.0–26.0)	1	1	
30–39	194/585	33.2 (31.0–35.4)	1.57 (1.09–2.26)^*^	1.75 (1.17–2.61)^**^	
40–49	165/565	29.2 (27.1–31.3)	1.30 (0.90–1.89)	1.50 (0.98–2.29)^†^	
50–59	131/338	38.8 (36.5–41.1)	2.00 (1.36–2.95)^***^	2.33 (1.48–3.67)^***^	
≥60	43/102	42.2 (39.9–44.5)	2.31 (1.39–3.83)^**^	2.59 (1.46–4.58)^**^	
Per 10 years increase (Continuous)	–	–	1.18 (1.08–1.29)^***^	1.22 (1.10–1.36)^***^	
Anxiety (*n* = 1820)					0.112
Age at diagnosis, years					
<30	77/206	37.4 (35.2–39.6)	1	1	
30–39	167/589	28.4 (26.3–30.5)	0.66 (0.47–0.93)^*^	0.66 (0.46–0.95)^*^	
40–49	192/583	32.9 (30.7–35.1)	0.82 (0.59–1.15)	0.86 (0.59–1.27)	
50–59	127/341	37.2 (35.0–39.4)	0.99 (0.70–1.42)	1.05 (0.69–1.59)	
≥60	42/101	41.6 (39.3–43.9)	1.19 (0.73–1.94)	1.29 (0.75–2.22)	
Per 10 years increase (Continuous)	–	–	1.11 (1.01–1.21)^*^	1.16 (1.04–1.29)^**^	
Sleep disorders (*n* = 1,419)					
Age at diagnosis, years					0.069
<30	92/159	57.9 (55.3–60.5)	1	1	
30–39	259/455	56.9 (54.3–59.5)	0.96 (0.67–1.39)	1.32 (0.87–2.01)	
40–49	305/456	66.9 (64.5–69.3)	1.47 (1.02–2.13)^*^	2.41 (1.55–3.75)^***^	
50–59	168/274	61.3 (58.8–63.8)	1.15 (0.78–1.72)	1.95 (1.21–3.14)^**^	
≥60	49/75	65.3 (62.8–67.8)	1.37 (0.78–2.43)	2.26 (1.18–4.31)^*^	
Per 10 years increase (Continuous)	–	–	1.11 (1.01–1.23)^*^	1.27 (1.12–1.43)^***^	

OR: Odds ratios; 95% CI: 95% confidence interval.

^aUnivariable model.^

^b^Adjusted for gender, BMI, living location, marital status, educational level, monthly family income, family history of cancer in first-relatives, smoking status, alcohol use, financial debt due to anti-cancer treatment, weekly frequency of physical activity, clinical stage, treatment modalities (intensity-modulated radiotherapy, chemotherapy, or surgery), status of distant metastasis and recurrence.

^†^*p* < 0.10; ^*^p < 0.05; ^**^p < 0.01; ^***^p < 0.001.

**Figure 1:**
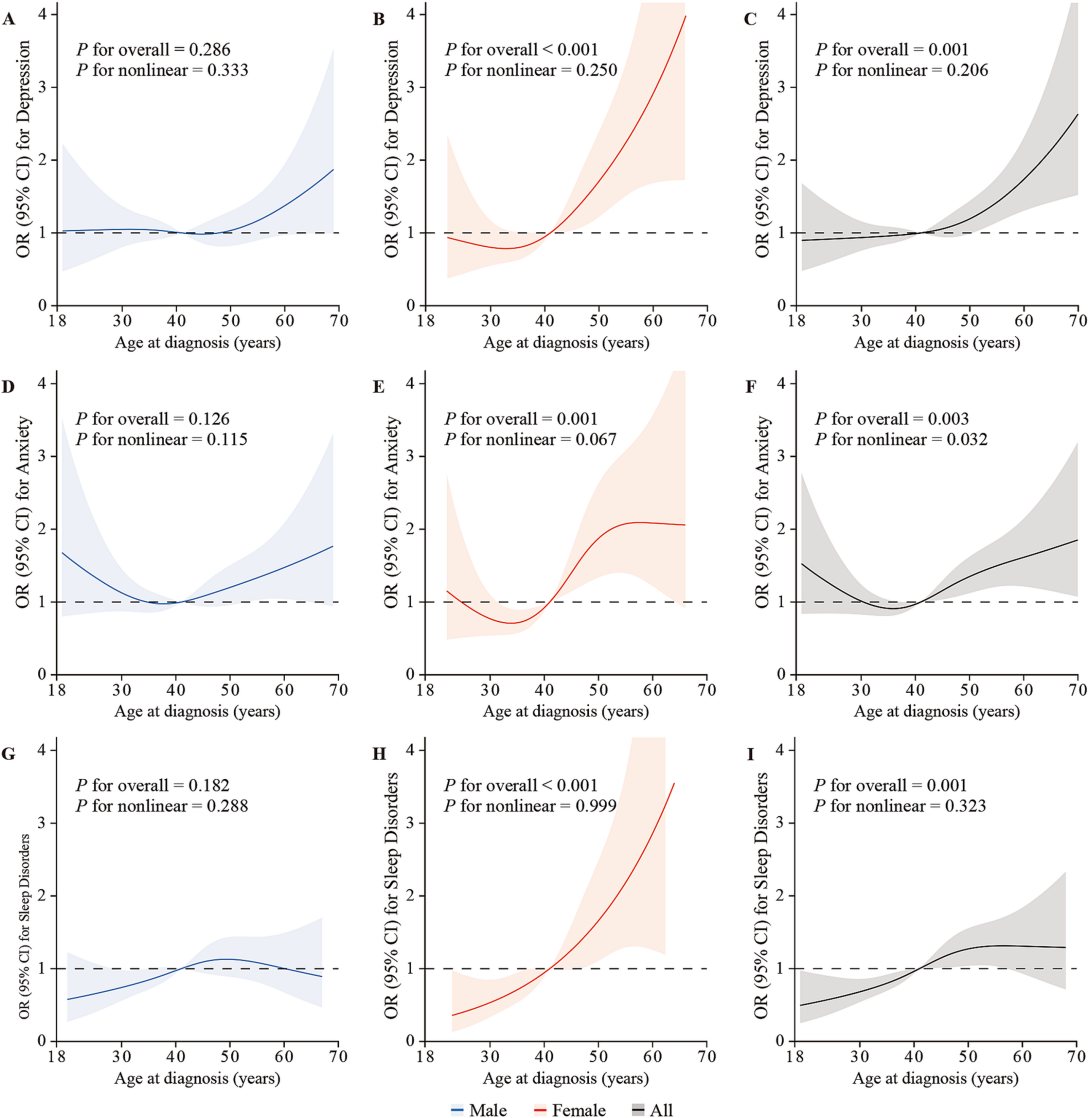
The restricted cubic splines depicting the associations between age at diagnosis and the risk of depression, anxiety, and sleep disorders via logistic regression in males **(A,D,G)**, females **(B,E,H)**, and overall **(C,F,I)**, adjusted for gender (only for overall analysis), age, BMI, living location, marital status, educational level, monthly family income, family history of cancer in first-relatives, financial debt due to anti-cancer treatment, smoking status, alcohol use, weekly frequency of physical activity, clinical stage, intensity-modulated radiotherapy, chemotherapy, surgery, distant metastasis, and recurrence. The lines represent the adjusted odds ratios with shaded bands indicating 95% confidence intervals. The median age at diagnosis (44 years) is set as the reference value. Knots were positioned at the 5th, 35th, 65th, and 95th percentiles of age at NPC diagnosis.

Overall, a nonlinear trend between age at diagnosis and the risk of anxiety was observed (*P*_nonlinear_ = 0.032), and the other nonlinear trends were statistically non-significant overall and by gender (all *P*_nonlinear_ > 0.05; Figure 1).

### Association between time since NPC diagnosis and mental disorders

Significantly negative associations were observed between time since NPC diagnosis and depression, anxiety, and sleep disorders. The aORs for depression were 0.52 (95% CI: 0.36–0.76; *p* = 0.001) for 6–11 months, 0.44 (95% CI: 0.31–0.62; *p* < 0.001) for 12–23 months, 0.52 (95% CI: 0.38–0.72; *p* < 0.001) for 24–59 months, and 0.58 (95% CI: 0.39–0.87; *p* = 0.008) for ≥60 months. The aORs for anxiety were 0.52 (95% CI: 0.36–0.75; *p* < 0.001) for 6–11 months, 0.49 (95% CI: 0.35–0.68; *p* < 0.001) for 12–23 months, 0.51 (95% CI: 0.38–0.70; *p* < 0.001) for 24–59 months, and 0.67 (95% CI: 0.46–0.98; *p* = 0.039) for ≥60 months. Regarding sleep disorders, the aORs were 0.62 (95% CI: 0.44–0.87; *p* = 0.006) for 24–59 months, and 0.47 (95% CI: 0.31–0.73; *p* = 0.001) for ≥60 months. Additionally, the aOR for every 12-month increase since diagnosis was 0.95 (95% CI: 0.91–0.98; *p* = 0.005) for sleep disorders. No interaction with gender was observed (Table 4). Similar associations were observed by gender (Supplementary Table 4).

**Table 4:** Association between time since diagnosis and depression, anxiety, and sleep disorders.

	Case/*n*	Prevalence (95% CI)	Crude OR (95% CI)^a^	Adjusted OR (95% CI)^b^	*P* for gender interaction
Depression (*n* = 1794)					
Time since diagnosis, months					0.199
<3	164/413	39.7 (37.4–42.0)	1	1	
3–5	104/234	44.4 (42.1–46.7)	1.21 (0.88–1.68)	1.11 (0.79–1.56)	
6–11	59/209	28.2 (26.1–30.3)	0.60 (0.42–0.86)^**^	0.52 (0.36–0.76)^**^	
12–23	76/320	23.8 (21.8–25.8)	0.47 (0.34–0.65)^***^	0.44 (0.31–0.62)^***^	
24–59	119/425	28.0 (25.9–30.1)	0.59 (0.44–0.79)^***^	0.52 (0.38–0.72)^***^	
≥60	60/193	31.1 (29.0–33.2)	0.68 (0.48–0.98)^*^	0.58 (0.39–0.87)^**^	
Per 6 months increase (Continuous)	–	–	0.99 (0.97–1.00)	0.98 (0.96–1.00)^†^	
Per 12 months increase (Continuous)	–	–	0.97 (0.94–1.01)	0.96 (0.93–1.00)^†^	
Anxiety (*n* = 1820)					
Time since diagnosis, months					0.635
<3	174/418	41.6 (39.3–43.9)	1	1	
3–5	97/237	40.9 (38.6–43.2)	0.97 (0.70–1.34)	0.93 (0.67–1.31)	
6–11	60/215	27.9 (25.8–30.0)	0.54 (0.38–0.77)^**^	0.52 (0.36–0.75)^***^	
12–23	85/324	26.2 (24.2–28.2)	0.50 (0.36–0.68)^***^	0.49 (0.35–0.68)^***^	
24–59	121/427	28.3 (26.2–30.4)	0.55 (0.42–0.74)^***^	0.51 (0.38–0.70)^***^	
≥60	68/199	34.2 (32.0–36.4)	0.73 (0.51–1.03)^†^	0.67 (0.46–0.98)^*^	
Per 6 months increase (Continuous)	–	–	0.99 (0.97–1.01)	0.99 (0.97–1.01)	
Per 12 months increase (Continuous)	–	–	0.98 (0.95–1.01)	0.98 (0.94–1.01)	
Sleep disorders (*n* = 1,419)					
Time since diagnosis, months					0.972
<3	209/311	67.2 (64.8–69.6)	1	1	
3–5	126/179	70.4 (68.0–72.8)	1.16 (0.78–1.73)	1.17 (0.77–1.77)	
6–11	104/165	63.0 (60.5–65.5)	0.83 (0.56–1.24)	0.78 (0.52–1.17)	
12–23	157/266	59.0 (56.4–61.6)	0.70 (0.50–0.99)^*^	0.71 (0.50–1.01)^†^	
24–59	196/345	56.8 (54.2–59.4)	0.64 (0.47–0.88)^**^	0.62 (0.44–0.87)^**^	
≥60	81/153	52.9 (50.3–55.5)	0.55 (0.37–0.82)^**^	0.47 (0.31–0.73)^**^	
Per 6 months increase (Continuous)	–	–	0.98 (0.96–1.00)^*^	0.97 (0.95–0.99)^**^	
Per 12 months increase (Continuous)	–	–	0.96 (0.92–0.99)^*^	0.95 (0.91–0.98)^**^	

OR: Odds ratios; 95% CI: 95% confidence interval.

^aUnivariable model.^

^b^Adjusted for gender, BMI, living location, marital status, educational level, monthly family income, family history of cancer in first-relatives, smoking status, alcohol use, financial debt due to anti-cancer treatment, weekly frequency of physical activity, clinical stage, treatment modalities (intensity-modulated radiotherapy, chemotherapy, or surgery), status of distant metastasis and recurrence.

^†^*p* < 0.10; ^*^p < 0.05; ^**^p < 0.01; ^***^p < 0.001.

**Figure 2:**
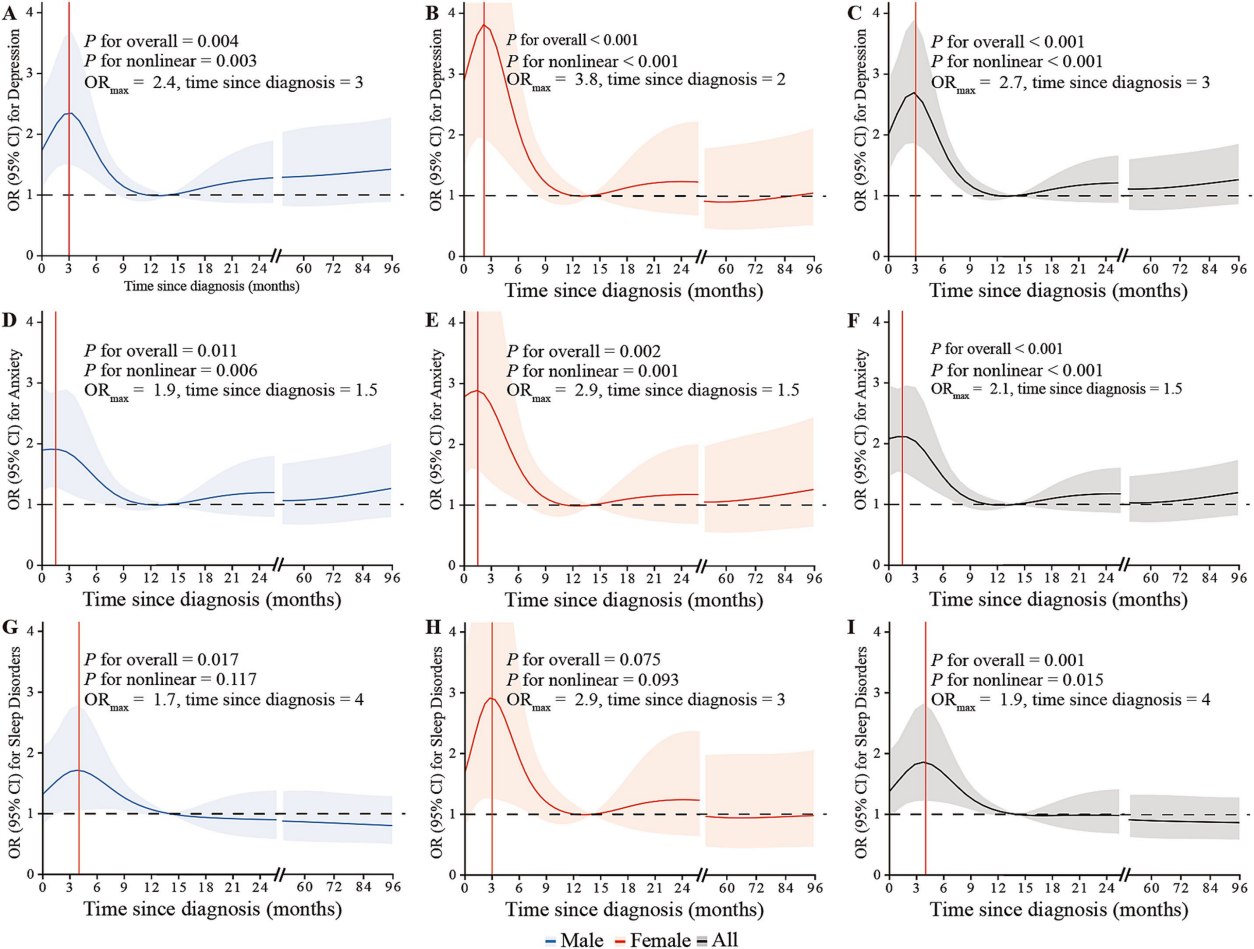
The restricted cubic splines depicting the associations between time since NPC diagnosis and the risk of depression, anxiety, and sleep disorders through logistic regression in males **(A,D,G)**, females **(B,E,H)**, and overall **(C,F,I)**, adjusted for gender, age, BMI, living location, marital status, educational level, monthly family income, family history of cancer in first-relatives, financial debt due to anti-cancer treatment, smoking status, alcohol use, weekly frequency of physical activity, clinical stage, intensity-modulated radiotherapy, chemotherapy, surgery, distant metastasis, and recurrence. The lines represent the adjusted odds ratios with shaded bands indicating 95% confidence intervals. The reference value is set at the median time since diagnosis (14 months). Knots are positioned at the 5th, 23rd, 41st, 59th, 77th, and 95th percentiles of time since NPC diagnosis.

A significant nonlinear trend of time since diagnosis with the risk of depression, anxiety, and sleep disorders was observed (all *P*_nonlinear_ < 0.05; Figure 2). The highest odds of depression, anxiety, and sleep disorders were observed at around 3.0 months (OR_max_: 2.7), 1.5 months (OR_max_: 2.1) and 4.0 (OR_max_: 1.9) months since NPC diagnosis, respectively, and then gradually recovered to a low-risk level at around 12 months.

## Discussion

This study represents the first comprehensive estimation of mental disorder prevalence among NPC patients in South China, one of the most significant NPC endemic regions globally, and also examines the associations between age at diagnosis and time since NPC diagnosis with depression, anxiety and sleep disorders. In this extensive cross-sectional study based on a large dataset, we discovered significantly high prevalences of depression, anxiety and sleep disorders among NPC patients, with common occurrences of comorbid mental disorders. The risk of these mental disorders showed a linear increase with age at diagnosis, except for anxiety. Additionally, a significant nonlinear trend was observed in the relationship between time since diagnosis and the risk of mental disorders. Specifically, the highest risk occurred within the first 4 months (ranging from 1.5 to 4.0 months) since NPC diagnosis, followed by a gradual decline to a general level around 12 months. Collectively, this study highlights the importance of raising awareness among healthcare professionals, family caregivers, and society as a whole regarding mental health issues in NPC survivors.

Similar to previous studies (11, 26), our findings showed that females were at a higher risk of mental disorders than males in NPC patients. This might be because females tend to be in a weaker social position, showing more vulnerability and a lower ability to cope with adverse events such as a cancer diagnosis (27). Therefore, it is crucial to pay more attention to females’ mental health issues during the diagnosis and treatment period of NPC. Our study also reveals that high physical activity and socioeconomic status were associated with low mental disorders among NPC patients, which aligns with prior studies (28). Previous studies in other cancer populations also suggested that higher levels of physical activity can alleviate symptoms of anxiety and depression (29, 30). These findings highlight the significance of incorporating physical activity programs as a part of comprehensive approaches to improve mental health in NPC patients.

In addition, our findings reveal that NPC patients diagnosed at an older age were more susceptible to mental disorders. However, reports regarding the associations between age at diagnosis and the risk of mental disorders vary among different cancer patients. Some studies on various cancer types (i.e., lung cancer, gynecological cancer, breast cancer, and colorectal cancer) have indicated that depression is more prevalent among younger patients (26, 31). Conversely, in patients with testicular germ cell tumors, a younger age at diagnosis and a shorter time since diagnosis were significantly associated with a higher risk of anxiety (32). Studies encompassing multiple cancer types have reported an association between younger age and poor sleep quality, as measured by self-report scales and structured clinical interviews (33–35). However, this association differs from that observed in NPC patients. A previous study in NPC patients reported similar results to our findings, indicating a positive association between poor sleep quality and older age before treatment (36). This difference in the association between depression, anxiety, and age at NPC diagnosis compared to other cancer types may be attributed in part to the specificity of NPC, including its favorable prognosis and age-specific mortality trends. It has been documented that age-specific mortalities of NPC in China start to increase rapidly from the ages of 35–39 years as of 2013 (6). Moreover, a younger age has been identified as an independent predictor of successful return to work, which is associated with a lower level of psychosocial burden and financial toxicity (37). Thus, older NPC patients, particularly females, should receive increased clinical attention and timely psychological support following NPC diagnosis.

We found nonlinear associations between the risk of mental disorders and time since NPC diagnosis, characterized by a sharp increase in risk within the first 3–4 months, followed by a gradual decrease to a low and stable level around 12 months after NPC diagnosis. A comparable trend between mental disorders and time since NPC diagnosis was documented in a longitudinal study, which indicated that the impact of NPC and its treatment on anxiety and depressive symptoms peaked at around 3 months after diagnosis (38). After cancer diagnosis, patients may experience a feeling of anticipatory grief, which is a forewarning or expecting the impending death (39, 40). As mentioned earlier, NPC patients have a favorable prognosis, and along with an increased understanding of NPC, their anticipatory grief may gradually lessen. The observed trend between mental disorders and time since NPC diagnosis may be partially attributed to variations in treatment stages. In our study, among patients with less than 3 months since NPC diagnosis, 18.6% were at the pre-treatment stage, 77.9% were undergoing treatment, and only 3.5% had completed anti-cancer treatment. For patients with 3–5 months since NPC diagnosis, 60.2% were undergoing treatment, and 39.8% had completed anti-cancer treatment. In patients with more than 6 months since NPC diagnosis, approximately 85% had completed anti-cancer treatment. The psychological well-being of NPC patients is influenced by the treatment stage. A nationwide population-based study reported that anxiety was more likely to be reported at the beginning of radiotherapy, while the proportion of depression increased after the start of treatment and peaked at 1 month post-treatment initiation, declining thereafter in NPC patients (41). The results from a prospective study revealed a significant increase in the proportion of NPC patients experiencing distress during the treatment period (42). One possible explanation is that NPC patients commonly endure severe acute side effects during treatment, which may further heighten the risk of mental disorders (43, 44). Another contributing factor could be the amplified financial burden resulting from treatment toxicity (28). Similarly, patients’ sleep quality correlates with the treatment stage. The severity of apnea and hypopnea events, as well as snoring, typically diminish in the majority of NPC patients 6 months after treatment (45). These findings suggest the importance of monitoring the mental health of NPC patients within the first year post-diagnosis, particularly during the initial 4 months. It is crucial to consider this monitoring in conjunction with the timing of reexaminations after diagnosis and treatment. This period typically encompasses the initiation of treatment and the subsequent follow-up after the first treatment. Providing timely psychological support during this high-risk period is highly warranted. It is recommended that oncologists acquire fundamental psychological knowledge to effectively conduct psychosocial assessments, enabling them to screen for mental health risks in cancer patients and provide timely interventions during this period. Additionally, collaboration with other relevant healthcare disciplines is vital. Cancer centers with the capacity should establish a referral system for psychiatric services, either within their facilities or in collaboration with external organizations.

However, several limitations of this study should be acknowledged. Firstly, the cross-sectional design prevented the assessment of longitudinal trends in depression, anxiety, and sleep disorders along with age at diagnosis and time since diagnosis. Therefore, prospective cohort studies are warranted to address this limitation. Secondly, although our study had a large sample size, the generalizability and representativeness of our findings should be cautious given that the sample was exclusively drawn from one cancer center in South China. The studies involving multiple centers from different regions are highly warranted. Thirdly, we did not evaluate the influence of mental disorders present before NPC diagnosis on our findings due to data limitations. Fourthly, the assessment of mental health conditions (i.e., depression, anxiety, and sleep disorders) relied on epidemiological screening instruments rather than structured diagnostic interviews, which may introduce potential misclassification despite the confirmed psychometric properties of these instruments. Lastly, the study was conducted during the COVID-19 pandemic, and the results may have been influenced by the pandemic.

## Conclusion

This study revealed a high level of mental disorders in NPC patients. The odds of these mental disorders increase linearly with age at diagnosis but display a nonlinear association with the time since diagnosis. The risk peaked around the first 3–4 months after NPC diagnosis, then gradually declined to a lower-risk level around 12 months. These findings are highly warranted for further confirm by large-scale longitudinal studies. The timely psychological support following the NPC diagnosis is of critical importance for improving the mental health of this population.

## Data Availability

The datasets presented in this study can be found in online repositories. The names of the repository/repositories and accession number(s) can be found below: the authenticity of this article has been validated by uploading the key raw data onto the Research Data Deposit platform (www.researchdata.org.cn) with the approval RDD number RDDA2024544536.

## Footnotes

^1^ www.researchdata.org.cn
