# Supplementary material for: Prevalence of mental disorders and their associations with age at diagnosis and time since diagnosis of nasopharyngeal cancer
Source: Front Public Health. 2024 Dec 3;12:1469001. doi: 10.3389/fpubh.2024.1469001 (PMC11653364; doi:10.3389/fpubh.2024.1469001)
Supplement: Supplementary file 1 [file Table_1.DOCX]

**Supplementary materials**

eTable 1. Univariate analysis of background factors related to depression, anxiety and sleep disorders.

eTable 2. The scores of HADS and PSQI with seven components.

eTable 3. Association between age at diagnosis and depression, anxiety, and sleep disorders by gender.

eTable 4. Association between time since diagnosis and depression, anxiety, and sleep disorders by gender

eFigure 1. Distribution of depression (A), anxiety (B), and sleep disorders (C) prevalence with age at NPC diagnosis.

eFigure 2. Distribution of depression (A), anxiety (B), and sleep disorders (C) prevalence with time since NPC diagnosis.

eTable 1. Univariate analysis of background factors related to depression, anxiety and sleep disorders.

| **Factors** | **Crude OR (95% CI) for depression** | **Crude OR (95% CI) for anxiety** | **Crude OR (95% CI) for sleep disorders** |
| --- | --- | --- | --- |
| **Social-demographic variables** |  |  |  |
| **Gender** |  |  |  |
| Male | 1 | 1 | 1 |
| Female | 1.18 (0.95 – 1.46) | 1.34 (1.08 – 1.65)^**^ | 1.69 (1.33 – 2.16)^***^ |
| **BMI, kg/m^2^** |  |  |  |
| 18.5 – 24.9 | 1 | 1 | 1 |
| <18.5 | 1.42 (1.08 – 1.87)^*^ | 1.27 (0.97 – 1.67)^†^ | 1.50 (1.08 – 2.08)^*^ |
| ≥25.0 | 0.84 (0.63 – 1.12) | 0.95 (0.72 – 1.25) | 0.98 (0.73 – 1.31) |
| **Living location** |  |  |  |
| Urban | 1 | 1 | 1 |
| Rural | 1.23 (1.00 – 1.50)^*^ | 0.98 (0.80 – 1.19) | 0.81 (0.65 – 1.01)^†^ |
| **Marital status** |  |  |  |
| Single (i.e., Unmarried/divorced/separated/widowed) | 1 | 1 | 1 |
| Married/cohabiting | 0.98 (0.74 – 1.30) | 0.92 (0.70 – 1.21) | 0.69 (0.50 – 0.96)^*^ |
| **Education level** |  |  |  |
| Primary school or below/Junior high school/High school or equivalent | 1 | 1 | 1 |
| Junior college/Bachelor's degree or above | 0.62 (0.50 – 0.77)^***^ | 0.82 (0.67 – 1.01)^†^ | 0.96 (0.77 – 1.20) |
| **Monthly family income, RMB** |  |  |  |
| <5000 | 1 | 1 | 1 |
| ≥5000 | 0.58 (0.47 – 0.71)^***^ | 0.78 (0.64 – 0.96)^*^ | 0.74 (0.60 – 0.92)^**^ |
| **Family history of cancer** |  |  |  |
| No | 1 | 1 | 1 |
| Yes | 1.20 (0.94 – 1.53) | 0.98 (0.77 – 1.26) | 1.11 (0.85 – 1.45) |
| **Financial debt due to anti-cancer treatment** |  |  |  |
| No | 1 | 1 | 1 |
| Yes | 1.42 (1.16 – 1.73)^**^ | 1.33 (1.10 – 1.62)^**^ | 1.16 (0.93 – 1.43) |
| **Smoking status** |  |  |  |
| Never | 1 | 1 | 1 |
| Former | 1.15 (0.93 – 1.43) | 1.03 (0.84 – 1.28) | 0.89 (0.71 – 1.13) |
| Current | 1.66 (1.03 – 2.67)^*^ | 1.22 (0.75 – 1.98) | 0.77 (0.46 – 1.28) |
| **Alcohol use** |  |  |  |
| No | 1 | 1 | 1 |
| Yes | 0.98 (0.80 – 1.21) | 0.83 (0.67 – 1.02)^†^ | 0.93 (0.74 – 1.17) |
| **Weekly frequency of physical activity** |  |  |  |
| Never | 1 | 1 | 1 |
| 1 time | 0.80 (0.60 – 1.07) | 0.80 (0.60 – 1.06) | 0.81 (0.58 – 1.13) |
| 2 – 3 times | 0.64 (0.48 – 0.85)^**^ | 0.77 (0.58 – 1.01)^†^ | 0.70 (0.51 – 0.97)^*^ |
| 4 – 5 times | 0.47 (0.31 – 0.71)^***^ | 0.45 (0.30 – 0.69)^***^ | 0.59 (0.39 – 0.90)^*^ |
| Almost everyday | 0.59 (0.44 – 0.80)^***^ | 0.67 (0.50 – 0.89)^**^ | 0.53 (0.38 – 0.73)^***^ |
| **Clinical variables** |  |  |  |
| **Clinical stage** |  |  |  |
| Ⅰ | 1 | 1 | 1 |
| Ⅱ | 1.87 (0.74 – 4.69) | 1.46 (0.62 – 3.43) | 0.96 (0.42 – 2.19) |
| Ⅲ | 2.55 (1.12 – 5.81)^*^ | 1.77 (0.83 – 3.77) | 0.95 (0.47 – 1.93) |
| Ⅳ | 2.40 (1.05 – 5.48)^*^ | 1.73 (0.81 – 3.69) | 1.04 (0.51 – 2.13) |
| **Age at diagnosis, years** |  |  |  |
| <30 | 1 | 1 | 1 |
| 30 – 39 | 1.57 (1.09 – 2.26)^*^ | 0.66 (0.47 – 0.93)^*^ | 0.96 (0.67 – 1.39) |
| 40 – 49 | 1.30 (0.90 – 1.89) | 0.82 (0.59 – 1.15) | 1.47 (1.02 – 2.13)^*^ |
| 50 – 59 | 2.00 (1.36 – 2.95)^***^ | 0.99 (0.70 – 1.42) | 1.15 (0.78 – 1.72) |
| ≥60 | 2.31 (1.39 – 3.83)^**^ | 1.19 (0.73 – 1.94) | 1.37 (0.78 – 2.43) |
| **Time since diagnosis, months** |  |  |  |
| <3 | 1 | 1 | 1 |
| 3 – 5 | 1.21 (0.88 – 1.68) | 0.97 (0.70 – 1.34) | 1.16 (0.78 – 1.73) |
| 6 – 11 | 0.60 (0.42 – 0.86)^**^ | 0.54 (0.38 – 0.77)^**^ | 0.83 (0.56 – 1.24) |
| 12 – 23 | 0.47 (0.34 – 0.65)^***^ | 0.50 (0.36 – 0.68)^***^ | 0.70 (0.50 – 0.99)^*^ |
| 24 – 59 | 0.59 (0.44 – 0.79)^***^ | 0.55 (0.42 – 0.74)^***^ | 0.64 (0.47 – 0.88)^**^ |
| ≥60 | 0.68 (0.48 – 0.98)^*^ | 0.73 (0.51 – 1.03)^†^ | 0.55 (0.37 – 0.82)^**^ |
| **Intensity-modulated radiotherapy** |  |  |  |
| No | 1 | 1 | 1 |
| Yes | 0.94 (0.63 – 1.42) | 0.89 (0.59 – 1.33) | 1.20 (0.77 – 1.86) |
| **Chemotherapy** |  |  |  |
| No | 1 | 1 | 1 |
| Yes | 1.61 (1.03 – 2.53)^*^ | 1.77 (1.12 – 2.79)^*^ | 1.08 (0.69 – 1.68) |
| **Surgery** |  |  |  |
| No | 1 | 1 | 1 |
| Yes | 1.26 (0.79 – 2.01) | 0.93 (0.57 – 1.50) | 1.36 (0.79 – 2.34) |
| **Distant metastasis** |  |  |  |
| No | 1 | 1 | 1 |
| Yes | 1.01 (0.76 – 1.33) | 0.96 (0.72 – 1.27) | 0.89 (0.66 – 1.21) |
| **Recurrence** |  |  |  |
| No | 1 | 1 | 1 |
| Yes | 1.41 (1.00 – 1.98) | 1.51 (1.07 – 2.12)^*^ | 1.74 (1.11 – 2.72)^*^ |

OR: Odds ratios; 95% CI: 95% confidence interval; BMI: Body mass index.

^†^*P*<0.10; ^*^*P*<0.05; ^**^*P*<0.01; ^***^*P*<0.001.

eTable 2. The scores of HADS and PSQI with seven components.

|  | **All** | | **Male** | | **Female** | | **t-test** |
| --- | --- | --- | --- | --- | --- | --- | --- |
|  | **Mean ± SD** | **Median [Min, Max]** | **Mean ± SD** | **Median [Min, Max]** | **Mean ± SD** | **Median [Min, Max]** | ***P* for gender** |
| HADS-D | 5.89 ± 3.79 | 6 [0, 21] | 5.75 ± 3.78 | 6 [0, 21] | 6.23 ± 3.78 | 6 [0, 18] | 0.016 |
| HADS-A | 6.00 ± 3.83 | 6 [0, 21] | 5.76 ± 3.83 | 6 [0, 21] | 6.57 ± 3.79 | 6 [0, 19] | <0.001 |
| Global PSQI score | 7.13 ± 3.73 | 6 [0, 20] | 6.84 ± 3.63 | 6 [0, 20] | 7.82 ± 3.86 | 7 [1, 20] | <0.001 |
| Subjective sleep quality | 1.30 ± 0.72 | 1 [0, 3] | 1.24 ± 0.71 | 1 [0, 3] | 1.43 ± 0.73 | 1 [0, 3] | <0.001 |
| Sleep latency | 1.17 ± 0.88 | 1 [0, 3] | 1.11 ± 0.86 | 1 [0, 3] | 1.30 ± 0.90 | 1 [0, 3] | <0.001 |
| Sleep duration | 1.04 ± 0.93 | 1 [0, 3] | 1.00 ± 0.91 | 1 [0, 3] | 1.14 ± 0.96 | 1 [0, 3] | 0.016 |
| Habitual sleep efficiency | 0.97 ± 1.06 | 1 [0, 3] | 0.90 ± 1.02 | 1 [0, 3] | 1.12 ± 1.13 | 1 [0, 3] | <0.001 |
| Sleep disturbances | 1.16 ± 0.59 | 1 [0, 3] | 1.14 ± 0.59 | 1 [0, 3] | 1.21 ± 0.60 | 1 [0, 3] | 0.054 |
| Use of sleep medications | 0.14 ± 0.56 | 0 [0, 3] | 0.13 ± 0.55 | 0 [0, 3] | 0.16 ± 0.58 | 0 [0, 3] | 0.407 |
| Daytime dysfunction | 1.35 ± 0.94 | 1 [0, 3] | 1.30 ± 0.93 | 1 [0, 3] | 1.47 ± 0.94 | 1 [0, 3] | 0.002 |

HADS-D: Hospital Anxiety and Depression Scale-Depression subscale; HADS-A: Hospital Anxiety and Depression Scale-Anxiety subscale; PSQI: Pittsburgh Sleep Quality Index; SD: standard deviation.

eTable 3. Association between age at diagnosis and depression, anxiety, and sleep disorders by gender.

|  | **Male** | | | | **Female** | | | |
| --- | --- | --- | --- | --- | --- | --- | --- | --- |
|  | **Case/*n*** | **Prevalence**  **(95% CI)** | **Crude OR**  **(95% CI)** *^a^* | **Adjusted OR**  **(95% CI)** *^b^* | **Case/*n*** | **Prevalence**  **(95% CI)** | **Crude OR**  **(95% CI)** *^a^* | **Adjusted OR**  **(95% CI)** *^b^* |
| **Depression** |  |  |  |  |  |  |  |  |
| **Age at diagnosis, years** |  |  |  |  |  |  |  |  |
| <30 | 34/137 | 24.8 (22.4 – 27.2) | 1 | 1 | 15/67 | 22.4 (18.8 – 26.0) | 1 | 1 |
| 30 – 39 | 138/405 | 34.1 (31.5 – 36.7) | 1.57 (1.01 – 2.43)^*^ | 1.56 (0.95 – 2.56) | 56/180 | 31.1 (27.2 – 35.0) | 1.57 (0.81 – 3.02) | 1.83 (0.89 – 3.78) |
| 40 – 49 | 114/413 | 27.6 (25.1 – 30.1) | 1.16 (0.74 – 1.80) | 1.17 (0.69 – 1.98) | 51/152 | 33.6 (29.6 – 37.6) | 1.75 (0.90 – 3.41) | 2.19 (1.03 – 4.65)^*^ |
| 50 – 59 | 84/237 | 35.4 (32.8 – 38.0) | 1.66 (1.04 – 2.66)^*^ | 1.68 (0.95 – 2.97) | 47/101 | 46.5 (42.2 – 50.8) | 3.02 (1.51 – 6.05)^**^ | 4.07 (1.84 – 9.03)^**^ |
| ≥60 | 27/73 | 37.0 (34.3 – 39.7) | 1.78 (0.96 – 3.28) | 1.68 (0.82 – 3.41) | 16/29 | 55.2 (51.0 – 59.4) | 4.27 (1.68 – 10.82)^**^ | 5.02 (1.79 – 14.12)^**^ |
| Per 10 years increase (Continuous) | -- | -- | 1.09 (0.98 – 1.22) | 1.09 (0.95 – 1.25) | -- | -- | 1.42 (1.20 – 1.68)^***^ | 1.51 (1.24 – 1.84)^***^ |
| **Anxiety** |  |  |  |  |  |  |  |  |
| **Age at diagnosis, years** |  |  |  |  |  |  |  |  |
| <30 | 55/139 | 39.6 (36.9 – 42.3) | 1 | 1 | 22/67 | 32.8 (28.8 – 36.8) | 1 | 1 |
| 30 – 39 | 112/411 | 27.3 (24.9 – 29.7) | 0.57 (0.38 – 0.86)^**^ | 0.55 (0.35 – 0.87)^*^ | 55/178 | 30.9 (27.0 – 34.8) | 0.91 (0.50 – 1.67) | 0.91 (0.47 – 1.75) |
| 40 – 49 | 126/424 | 29.7 (27.2 – 32.2) | 0.65 (0.43 – 0.96)^*^ | 0.65 (0.40 – 1.05) | 66/159 | 41.5 (37.3 – 45.7) | 1.45 (0.80 – 2.64) | 1.66 (0.84 – 3.28) |
| 50 – 59 | 79/238 | 33.2 (30.6 – 35.8) | 0.76 (0.49 – 1.17) | 0.76 (0.45 – 1.28) | 48/103 | 46.6 (42.4 – 50.8) | 1.79 (0.94 – 3.39) | 2.29 (1.10 – 4.77)^*^ |
| ≥60 | 30/72 | 41.7 (39.0 – 44.4) | 1.09 (0.61 – 1.95) | 1.08 (0.55 – 2.11) | 12/29 | 41.4 (37.2 – 45.6) | 1.44 (0.59 – 3.54) | 1.92 (0.71 – 5.20) |
| Per 10 years increase (Continuous) | -- | -- | 1.05 (0.94 – 1.17) | 1.08 (0.95 – 1.24) | -- | -- | 1.25 (1.06 – 1.47)^**^ | 1.38 (1.14 – 1.67)^**^ |
| **Sleep disorders** |  |  |  |  |  |  |  |  |
| **Age at diagnosis, years** |  |  |  |  |  |  |  |  |
| <30 | 63/111 | 56.8 (53.7 – 59.9) | 1 | 1 | 29/48 | 60.4 (55.8 – 65.0) | 1 | 1 |
| 30 – 39 | 166/310 | 53.5 (50.4 – 56.6) | 0.88 (0.57 – 1.36) | 1.07 (0.65 – 1.77) | 93/145 | 64.1 (59.5 – 68.7) | 1.17 (0.60 – 2.29) | 2.69 (1.18 – 6.14)^*^ |
| 40 – 49 | 214/330 | 64.8 (61.8 – 67.8) | 1.41 (0.91 – 2.18) | 1.99 (1.17 – 3.39)^*^ | 91/126 | 72.2 (67.9 – 76.5) | 1.70 (0.85 – 3.42) | 4.28 (1.79 – 10.23)^**^ |
| 50 – 59 | 103/190 | 54.2 (51.1 – 57.3) | 0.90 (0.56 – 1.45) | 1.33 (0.74 – 2.37) | 65/84 | 77.4 (73.4 – 81.4) | 2.24 (1.04 – 4.85)^*^ | 6.07 (2.29 – 16.05)^***^ |
| ≥60 | 29/52 | 55.8 (52.7 – 58.9) | 0.96 (0.49 – 1.87) | 1.32 (0.61 – 2.85) | 20/23 | 87.0 (83.8 – 90.2) | 4.37 (1.14 – 16.75)^*^ | 12.18 (2.66 – 55.9)^**^ |
| Per 10 years increase (Continuous) | -- | -- | 1.03 (0.92 – 1.17) | 1.12 (0.97 – 1.30) | -- | -- | 1.37 (1.12 – 1.68)^**^ | 1.75 (1.35 – 2.28)^***^ |

OR: Odds ratios; 95% CI: 95% confidence interval.

*^a^* Univariable model.

*^b^* Adjusted for BMI, living location, marital status, educational level, monthly family income, family history of cancer in first-relatives, smoking status, alcohol use, financial debt due to anti-cancer treatment, weekly frequency of physical activity, clinical stage, treatment modalities (intensity-modulated radiotherapy, chemotherapy, or surgery), status of distant metastasis and recurrence.

^†^*P*<0.10; ^*^*P*<0.05; ^**^*P*<0.01; ^***^*P*<0.001.

eTable 4. Association between time since diagnosis and depression, anxiety, and sleep disorders by gender

|  | **Male** | | | | **Female** | | | |
| --- | --- | --- | --- | --- | --- | --- | --- | --- |
|  | **Case/*n*** | **Prevalence**  **(95% CI)** | **Crude OR**  **(95% CI)** *^a^* | **Adjusted OR**  **(95% CI)** *^b^* | **Case/*n*** | **Prevalence**  **(95% CI)** | **Crude OR**  **(95% CI)** *^a^* | **Adjusted OR**  **(95% CI)** *^b^* |
| **Depression** |  |  |  |  |  |  |  |  |
| **Time since diagnosis, months** |  |  |  |  |  |  |  |  |
| <3 | 100/282 | 35.5 (32.9 – 38.1) | 1 | 1 | 64/131 | 48.9 (44.6 – 53.2) | 1 | 1 |
| 3 – 5 | 74/172 | 43.0 (40.3 – 45.7) | 1.37 (0.93 – 2.03) | 1.23 (0.82 – 1.86) | 30/62 | 48.4 (44.1 – 52.7) | 0.98 (0.54 – 1.80) | 0.91 (0.48 – 1.72) |
| 6 – 11 | 39/145 | 26.9 (24.5 – 29.3) | 0.67 (0.43 – 1.04) | 0.57 (0.36 – 0.92)^*^ | 20/64 | 31.2 (27.3 – 35.1) | 0.48 (0.25 – 0.89)^*^ | 0.42 (0.22 – 0.81)^*^ |
| 12 – 23 | 52/224 | 23.2 (20.9 – 25.5) | 0.55 (0.37 – 0.82)^**^ | 0.53 (0.35 – 0.80)^**^ | 24/96 | 25.0 (21.3 – 28.7) | 0.35 (0.20 – 0.62)^***^ | 0.31 (0.16 – 0.58)^***^ |
| 24 – 59 | 90/305 | 29.5 (27.0 – 32.0) | 0.76 (0.54 – 1.08) | 0.71 (0.49 – 1.04) | 29/120 | 24.2 (20.6 – 27.8) | 0.33 (0.19 – 0.57)^***^ | 0.28 (0.15 – 0.50)^***^ |
| ≥60 | 42/137 | 30.7 (28.2 – 33.2) | 0.80 (0.52 – 1.25) | 0.76 (0.46 – 1.24) | 18/56 | 32.1 (28.1 – 36.1) | 0.50 (0.26 – 0.96)^*^ | 0.37 (0.18 – 0.76)^**^ |
| Per 6 months increase (Continuous) | -- | -- | 0.99 (0.97 – 1.01) | 0.99 (0.96 – 1.01) | -- | -- | 0.98 (0.95 – 1.01) | 0.97 (0.94 – 1.00)^†^ |
| Per 12 months increase (Continuous) | -- | -- | 0.98 (0.93 – 1.02) | 0.98 (0.93 – 1.03) | -- | -- | 0.96 (0.90 – 1.03) | 0.94 (0.88 – 1.01)^†^ |
| **Anxiety** |  |  |  |  |  |  |  |  |
| **Time since diagnosis, months** |  |  |  |  |  |  |  |  |
| <3 | 107/285 | 37.5 (34.9 – 40.1) | 1 | 1 | 67/133 | 50.4 (46.2 – 54.6) | 1 | 1 |
| 3 – 5 | 68/173 | 39.3 (36.6 – 42.0) | 1.08 (0.73 – 1.59) | 1.01 (0.67 – 1.51) | 29/64 | 45.3 (41.1 – 49.5) | 0.82 (0.45 – 1.48) | 0.80 (0.42 – 1.49) |
| 6 – 11 | 41/148 | 27.7 (25.3 – 30.1) | 0.64 (0.41 – 0.98)^*^ | 0.63 (0.40 – 0.99)^*^ | 19/67 | 28.4 (24.6 – 32.2) | 0.39 (0.21 – 0.73)^**^ | 0.34 (0.17 – 0.65)^**^ |
| 12 – 23 | 55/230 | 23.9 (21.6 – 26.2) | 0.52 (0.36 – 0.77)^**^ | 0.53 (0.36 – 0.80)^**^ | 30/94 | 23.8 (20.2 – 27.4) | 0.46 (0.27 – 0.80)^**^ | 0.41 (0.23 – 0.76)^**^ |
| 24 – 59 | 86/306 | 28.1 (25.6 – 30.6) | 0.65 (0.46 – 0.92)^*^ | 0.63 (0.43 – 0.92)^*^ | 35/121 | 28.9 (25.1 – 32.7) | 0.40 (0.24 – 0.67)^**^ | 0.31 (0.18 – 0.55)^***^ |
| ≥60 | 45/142 | 31.7 (29.2 – 34.2) | 0.77 (0.50 – 1.18) | 0.76 (0.47 – 1.22) | 23/57 | 40.4 (36.2 – 44.6) | 0.67 (0.36 – 1.25) | 0.58 (0.30 – 1.14) |
| Per 6 months increase (Continuous) | -- | -- | 0.99 (0.97 – 1.01) | 0.99 (0.97 – 1.02) | -- | -- | 0.99 (0.96 – 1.02) | 0.99 (0.96 – 1.02) |
| Per 12 months increase (Continuous) | -- | -- | 0.98 (0.94 – 1.02) | 0.98 (0.94 – 1.03) | -- | -- | 0.98 (0.93 – 1.04) | 0.97 (0.92 – 1.03) |
| **Sleep disorders** |  |  |  |  |  |  |  |  |
| **Time since diagnosis, months** |  |  |  |  |  |  |  |  |
| <3 | 132/208 | 63.5 (60.5 – 66.5) | 1 | 1 | 77/103 | 74.8 (70.7 – 78.9) | 1 | 1 |
| 3 – 5 | 91/133 | 68.4 (65.5 – 71.3) | 1.25 (0.79 – 1.98) | 1.28 (0.79 – 2.07) | 35/46 | 76.1 (72.1 – 80.1) | 1.07 (0.48 – 2.42) | 0.95 (0.40 – 2.24) |
| 6 – 11 | 62/107 | 57.9 (54.8 – 61.0) | 0.79 (0.49 – 1.28) | 0.78 (0.47 – 1.28) | 42/58 | 72.4 (68.2 – 76.6) | 0.89 (0.43 – 1.83) | 0.70 (0.32 – 1.50) |
| 12 – 23 | 106/190 | 55.8 (52.7 – 58.9) | 0.73 (0.49 – 1.09) | 0.74 (0.49 – 1.14) | 51/76 | 67.1 (62.6 – 71.6) | 0.69 (0.36 – 1.32) | 0.52 (0.26 – 1.07) |
| 24 – 59 | 129/246 | 52.4 (49.3 – 55.5) | 0.63 (0.44 – 0.93)^*^ | 0.61 (0.41 – 0.92)^*^ | 67/99 | 67.7 (63.3 – 72.1) | 0.71 (0.38 – 1.30) | 0.52 (0.27 – 1.03) |
| ≥60 | 55/109 | 50.5 (47.4 – 53.6) | 0.59 (0.37 – 0.94)^*^ | 0.48 (0.29 – 0.81)^**^ | 26/44 | 59.1 (54.4 – 63.8) | 0.49 (0.23 – 1.03) | 0.39 (0.17 – 0.88)^*^ |
| Per 6 months increase (Continuous ) | -- | -- | 0.97 (0.95 – 1.00)^*^ | 0.97 (0.94 – 0.99)^*^ | -- | -- | 0.99 (0.96 – 1.02) | 0.98 (0.94 – 1.01) |
| Per 12 months increase (Continuous) | -- | -- | 0.95 (0.91 – 0.99)^*^ | 0.94 (0.89 – 0.99)^*^ | -- | -- | 0.97 (0.92 – 1.03) | 0.95 (0.89 – 1.02) |

OR: Odds ratios; 95% CI: 95% confidence interval.

*^a^* Univariable model.

*^b^* Adjusted for BMI, living location, marital status, educational level, monthly family income, family history of cancer in first-relatives, smoking status, **alcohol use**, **financial debt** due to anti-cancer treatment, weekly frequency of physical activity, clinical stage, treatment modalities (intensity-modulated radiotherapy, chemotherapy, or surgery), status of distant metastasis and recurrence.

^†^*P*<0.10; ^*^*P*<0.05; ^**^*P*<0.01; ^***^*P*<0.001.


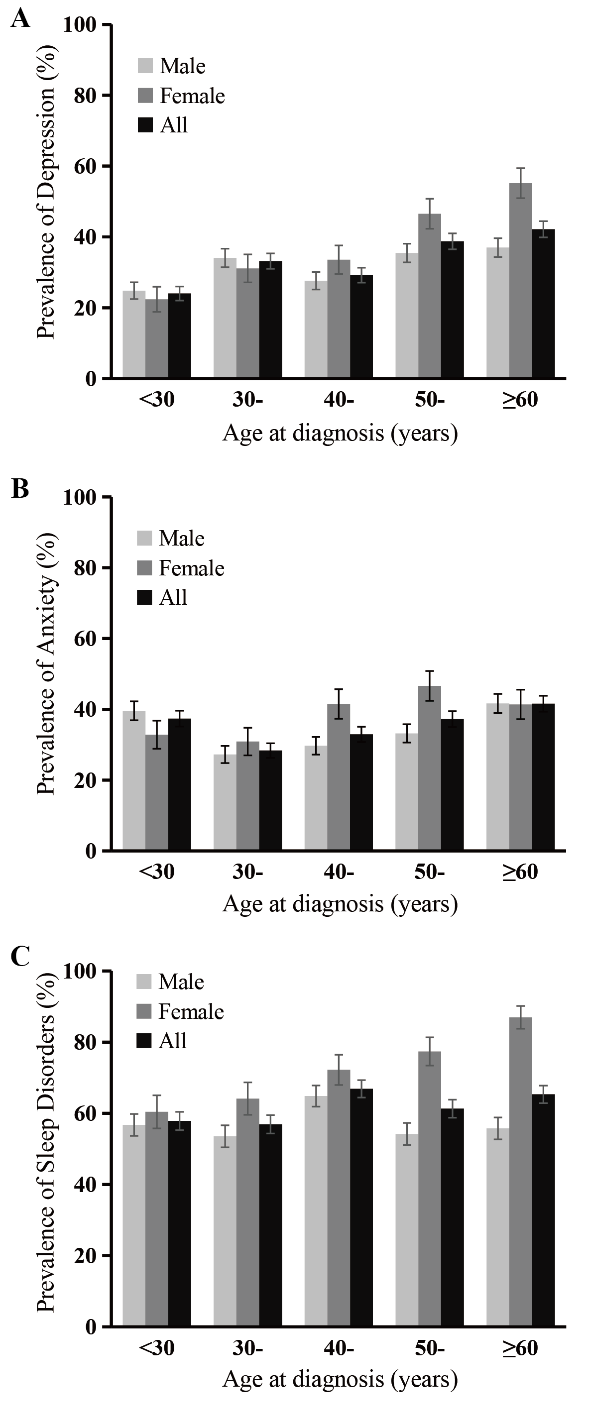


eFigure 1. Distribution of depression (A), anxiety (B), and sleep disorders (C) prevalence with age at NPC diagnosis.


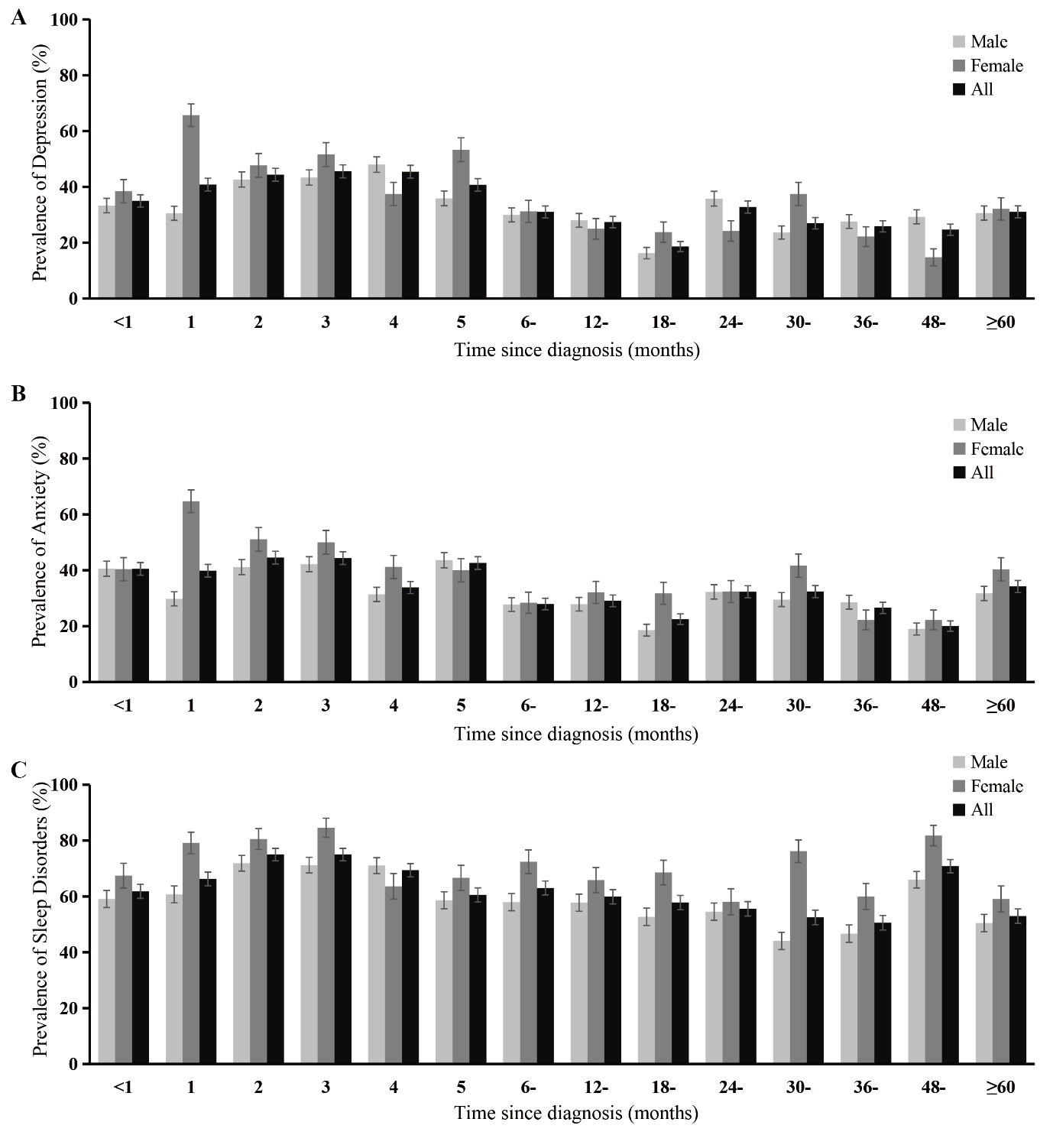


eFigure 2. Distribution of depression (A), anxiety (B), and sleep disorders (C) prevalence with time since NPC diagnosis.
